# Optimized extraction and analysis methods using liquid chromatography-tandem mass spectrometry for zearalenone and metabolites in human placental tissue

**DOI:** 10.1016/j.heliyon.2023.e16940

**Published:** 2023-06-04

**Authors:** Abigail Lazofsky, Anita Brinker, Ruby Gupta, Emily Barrett, Lauren M. Aleksunes, Zorimar Rivera-Núñez, Brian Buckley

**Affiliations:** a Environmental and Occupational Health Sciences Institute, Rutgers University, 170 Frelinghuysen Road, Piscataway, NJ, 08854, USA; b Department of Environmental and Occupational Health and Justice, Rutgers School of Public Health, Rutgers University, 683 Hoes Lane West, Piscataway, NJ, 08854, USA; c Department of Biostatistics and Epidemiology, Rutgers School of Public Health, Rutgers University, 683 Hoes Lane West, Piscataway, NJ, 08854, USA; d Department of Pharmacology and Toxicology, Ernest Mario School of Pharmacy, 160 Frelinghuysen Road, Rutgers University, Piscataway, NJ, 08854, USA; e Rutgers Center for Lipid Research, New Jersey Institute for Food, Nutrition, and Health, Rutgers University, 61 Dudley Road, New Brunswick, NJ, 08901, USA

**Keywords:** Zeranols, Mycotoxins, Mycoestrogens, Placenta, Biomarker, Solid-phase extraction

## Abstract

Zearalenone and its metabolites, a group of endocrine disrupting mycotoxins, have been linked to adverse reproductive health effects. They cross the placental barrier, potentially reaching the fetus. In this study, we adapted and optimized our protocol previously used for urine, to measure these mycotoxins in human placentas. We combined a supported liquid extraction step using Chem Elut cartridges with solid phase extraction on Discovery® DSC-NH2 tubes. The optimized extraction efficiencies were between 68 and 80% for all metabolites. Analysis was performed by UHPLC-HRMS using a Betasil™ Phenyl-Hexyl column eluted with a gradient of acetonitrile-methanol-water. The chromatography method separated all analytes in under 15 min. Validation experiments confirmed the method's sensitivity, with LODs ranging from 0.0055 to 0.011 pg/mg tissue. The method was linear over a range of 0.0025–1.5 pg/mg tissue with R^2^ values ≥ 0.994. Precision and accuracy calculations ranged from 4.7–7.9% and 0.6–6.7% respectively. The method was then successfully applied to a subset of placenta samples (n = 25) collected from an ongoing prospective birth cohort. Interestingly, 92% of the samples contained at least one measurable zearalenone metabolite, providing initial indication of potentially widespread exposure during pregnancy.

## Highlights

• Zearalenone and metabolites cross the placenta and can impact fetal development.
• First protocol to use Solid Phase Extraction for mycoestrogens in human placenta.
• Optimized Solid Phase Extraction method resulted in recoveries of 68–80%.
• Validated LC-MS method had low LODs of 0.0055–0.011 pg/mg tissue.
• Quantification of at least one metabolite in 92% of placenta samples (n = 25).

## Graphical abstract

**Figure:**
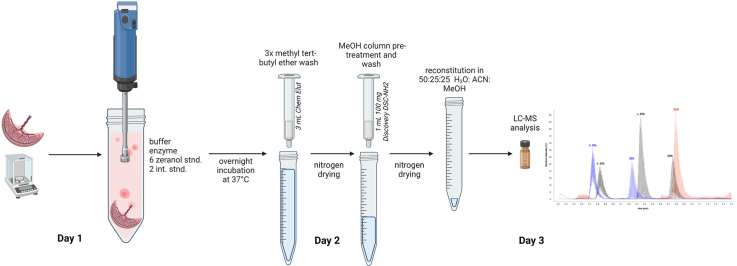

## 1 Introduction

Zearalenone (ZEN) is one of the most common naturally-occurring mycotoxins contaminating global food supplies [1]. A synthetic version of its metabolite, zeranol (α-zearalanol, ZER), is widely used as an anabolic growth promoter in U.S. livestock, though it has been banned in many countries including those in the European Union [2]. ZEN and ZER are present in a wide range of food products including cereal grains, meat, milk, wine, beer, dried fruit, and spices [[3], [4], [5]]. It is expected that human dietary exposure to ZEN will increase as global temperatures rise, and together with moisture, will likely create ideal conditions for *Fusarium* fungi to flourish within grain products [1]. Both mycotoxins and their metabolites (α-zearalenol (α-ZOL), β-zearalenol (β-ZOL), β-zearalanol (β-ZAL), and zearalanone (ZAN)) bind to and alter signaling of estrogen receptors α and β [[6], [7], [8], [9]] leading to their characterization as mycoestrogens. In animal models, ZEN and ZER exposure alters offspring development, resulting in earlier vaginal opening, lower body weight, shortened anogenital distance (AGD), and decreased serum estradiol [[10], [11], [12], [13], [14], [15], [16]]. A recent systematic review of in vitro and in vivo studies reported compelling evidence that mycoestrogen exposure affects female reproductive organs and hormone activity [17].

Less is known about the adverse impacts of mycoestrogens in humans. In two small clinical studies, ZEN exposure was associated with premature thelarche and precocious puberty [18,19]. In contrast, our group has reported associations between prepubertal exposure to ZEN and slower growth and pubertal development in girls [20,21]. However, ZEN impact in humans during the prenatal period, a hypothesized critical window of exposure, is unknown. In humans, ZEN crosses the placenta, a critical organ for fetal development and growth [16,22,23]. Quantifying mycoestrogens in human placenta is important because it may provide a better, more proximate characterization of fetal exposure than maternal measurements in urine or serum.

Mycoestrogens have been measured in serum, plasma, and urine from human populations [18,[24], [25], [26], [27]]. Solid Phase Extraction (SPE) is often the utilitarian first step for the isolation of analytes from complex biological matrices. SPE combines quantitative extraction and cleanup, which can increase recovery yields in reduced preparation time [28]. There are limited reported research studies using SPE for the pretreatment of placental tissues. Two animal studies examining fetal exposure to ZEN, α-ZOL, and β-ZOL in rats [29] and ZER in rabbits [30] included SPE as part of their sample extraction protocol with successful results.

Previously, our research group quantified ZEN and its metabolites from mouse tissue (including placenta) [31] as well as human urine and serum [20,21,32]. The aim of this study was to optimize and adapt our current sample preparation and analysis protocols for human placental tissue to measure ZEN and its metabolites.

## 2 Materials and methods

### 2.1 Placenta collection and storage

#### 2.1.1 Placentas collected for method development

Placentas were collected from women undergoing scheduled cesarean deliveries at Robert Wood Johnson University Hospital in New Brunswick, NJ. Participant inclusion criteria for this study were healthy women, ages 18–40, and term gestation. Exclusion criteria were pregnancy-induced medical conditions (i.e., pregnancy-induced hypertension, preeclampsia, gestational diabetes), chronic medical disorders (i.e., hypertension, diabetes, autoimmune diseases), maternal infection, clinical chorioamnionitis, medication use (except prenatal vitamins), maternal alcohol or drug use, and known fetal chromosomal abnormalities. Placentas were collected within 10 min of delivery for processing within 1 h. Visible abnormalities and the location of the umbilical cord were assessed and only normal placentas with central or eccentric cord insertions were used. To sample placental tissue, the overlying membranes, maternal decidua, and chorionic plate were removed. From the maternal side of the placenta, two pieces of chorionic tissue were collected along the long axis approximately 1 cm distal to the cord insertion site. Samples were snap frozen in liquid nitrogen and stored in a −80 °C freezer. This protocol was approved by the Rutgers University Institutional Review Board (IRB).

#### 2.1.2 Placentas demonstrating method application

Placentas were collected from women enrolled in the Understanding Pregnancy Signals and Infant Development (UPSIDE) study, an ongoing prospective birth cohort originally designed to study maternal prenatal psychological distress and child outcomes in Rochester, NY [33]. Inclusion criteria were: 1) singleton pregnancies, 2) ≥18 years old, 3) no known substance abuse problems or history of psychotic illness, 4) ability to communicate in English. Additionally, women with major endocrine conditions were excluded (e.g., polycystic ovary syndrome). The research protocol was approved by the IRBs of the University of Rochester School of Medicine and Dentistry and Rutgers University. All study activities were explained to participants before obtaining written consent to participate in the study.

### 2.2 Chemicals and media

HPLC grade water, acetonitrile (ACN), and methanol (MeOH) were purchased from Thomas Scientific (Swedesboro, NJ, USA) and Sigma-Aldrich (St. Louis, MO, USA). Water from a Milli-Q® water purification system (MilliporeSigma, Burlington, MA, USA) was also used. HPLC grade methyl *tert*-butyl ether (MTBE) was purchased from VWR (Radner, PA, USA). Sodium acetate buffer (pH 4.65, 0.2 M) was obtained from Fluka (Honeywell Products, Charlotte, NC, USA). β-Glucuronidase from *Helix pomatia* (type HP-2, >100000 units/mL) was purchased from Sigma-Aldrich. ZEN, ZER, α-ZOL, β-ZOL, β-ZAL, ZAN standards were all purchased from Sigma-Aldrich. Internal standards Rac-Zearalenone-d_6_ (ZEN-d_6_; used for ZAN and ZEN) and α-Zearalenol-d_7_ (α-ZOL-d_7_; used for the other compounds) were obtained from Toronto Research Chemicals (North York, ON, Canada). Chem Elut supported liquid extraction (SLE) columns (3 mL, unbuffered) were from Agilent (Santa Clara, CA, USA); Discovery DSC-NH2 solid-phase extraction tubes (1 mL/100 mg) were from Supelco (Bellefonte, PA, USA); Sep-Pak silica cartridges (3 cc/500 mg) were from Waters (Milford, MA, USA).

### 2.3 Stock standard solutions

Stock solutions were individually prepared by dissolving 1 mg of pure compound in ACN. 100 μL of 1 mg/mL stock standard solutions of ZEN, ZER, α-ZOL, β-ZOL, β-ZAL, ZAN were combined and diluted with ACN to make 1 mL of a 100 μg/mL zeranol standard. The mixture was serially diluted to create the following spiking concentrations: 0.025, 0.05, 0.1, 0.5, and 2.5 ng/mL. Similarly, 100 μL of 1 mg/mL stock standard solutions of ZEN-d6 and αZOL-d7 were combined and diluted to make 1 mL of a 100 μg/mL internal standard (IS) mixture, which was then further diluted to make an internal standard spiking solution with 0.5 ng/mL of each internal standard. Solutions were stored in amber glass vials at −20 °C. Matrix matched standards were created by spiking the appropriate concentration of the diluted stock standards in homogenized tissue.

### 2.4 Sample preparation

**Fig. 1:**
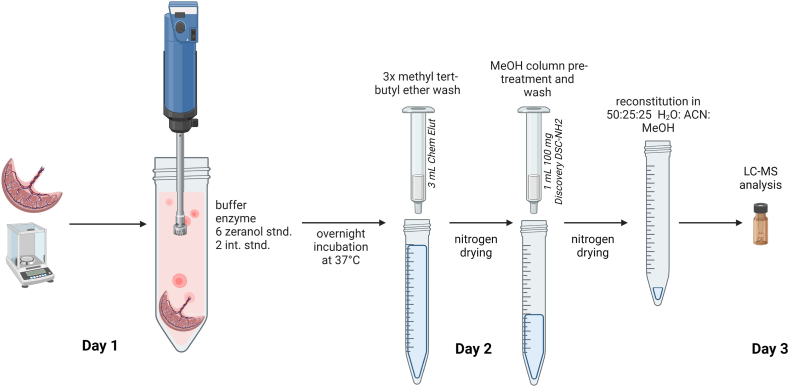
Sample preparation protocol overview for the extraction and analysis of mycoestrogens in placenta. The tissue samples were homogenized to form a fluid-like slurry, which was then carried through the extraction procedure. The mycoestrogens standard mixture is only added to placental tissues used for the creation of the calibration curve.

An overview of the final sample preparation protocol is shown in Fig. 1; it is a modification of our method used to analyze ZEN and its metabolites in urine [21]. Frozen placenta tissue was cut, weighed (approx. 200 mg), and placed into 50 mL polypropylene centrifuge tubes (VWR). Sodium acetate buffer (1 mL) was added, and the samples were homogenized with a Tissue Tearor (BioSpec Products, Bartlesville, OK, USA) until there was no visually intact tissue present in the mixture to form a slurry. The homogenizer probe was then rinsed once with 1 mL buffer and the rinsate was added to the sample. For the matrix matched standard curve, approximately 1.75 g of tissue that was previously found to have low levels of the analytes was homogenized with 12 mL buffer; the homogenizer probe was rinsed with 2 mL buffer that was added to the homogenate. This homogenate slurry was aliquoted to 50 mL tubes to give the equivalent of approximately 200 mg tissue per tube, and 25 μL of the zeranols standard mixture at various concentrations (see *above*) was added. 25 μL of the IS mixture (0.5 ng/mL each standard) and 10 μL of β-glucuronidase (to hydrolyze conjugated forms of the analytes and measure total zeranols) were added to each sample and matrix matched standard. A blank sample with only buffer and the enzyme was also prepared. The tubes were incubated overnight in a shaking water bath (30 RPM) at 37 °C and were then centrifuged at 1500 RPM for 5 min to bring down liquid that had condensed on the sides.

Following centrifugation, the slurry samples were added to Chem Elut columns; vacuum was applied until all the liquid had been pulled into the packing. The columns were then eluted with 3 × 5 mL MTBE (15 mL total). A vacuum was used as necessary to pull eluent through the column. MTBE is less dense than the aqueous sample, so all sample liquid was pulled onto the column before application of the MTBE wash. Therefore, it was necessary to put the columns under vacuum after the samples were applied until the samples had completely passed into the columns, sometimes for a half hour or more. The eluates from the Chem Elut columns were dried under nitrogen and then redissolved in 0.5 mL MeOH. Extracts were then loaded onto Discovery DSC-NH2 SPE columns that had been equilibrated with 0.5 mL MeOH; the analytes were eluted with 0.5 mL MeOH and dried under nitrogen. Finally, the extracts were redissolved in 50 μL of the initial HPLC solvent (50% water, 25% ACN, 25% MeOH), transferred to amber glass LC vials with inserts, and stored at −20 °C until analysis.

### 2.5 Liquid chromatography mass spectrometry (LCMS) conditions

**Fig. 2:**
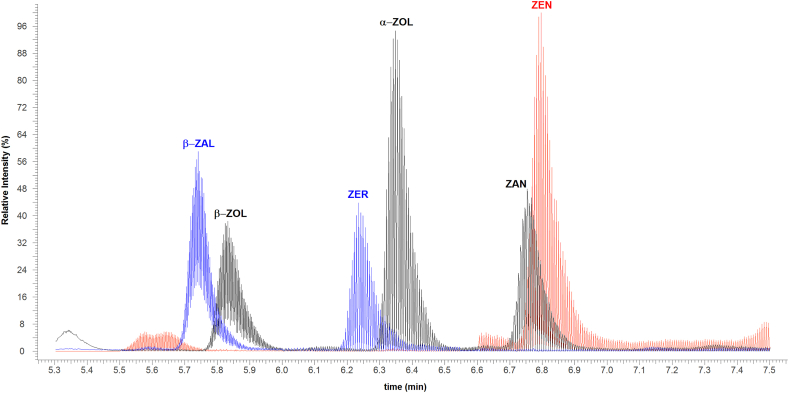
Chromatogram of all mycoestrogens, as observed in a spiked-placenta standard at 5 pg/mg tissue.

Separation and analysis were performed using a Dionex UltiMate 3000 UHPLC interfaced to a Thermo Scientific Q Exactive HF Hybrid Quadrupole-Orbitrap equipped with a HESI-II electrospray ionization (ESI) probe (Thermo Fisher Scientific, Waltham, MA). Chromatographic separation was performed using a BetaSil™ phenyl hexyl column (100 × 4.6 mm, 3 μm) from Thermo Fisher Scientific at 35 °C. The mobile phase was comprised of water (solvent A), MeOH (solvent B), and ACN (solvent C). The gradient was as follows: 0–0.5 min 25% B and C each; 0.5–3 min linear ramp to 40% B and C each; 3–5 min linear ramp to 47.5% B and C each, 5–11 min 47.5% B and C each; 11–11.01 min linear ramp to 25% B and C each, equilibrate for 4 min prior to the next analysis (15 min total). A diverter valve redirected the eluate to waste for the last half of the run. The flow rate was 0.5 mL/min and the injection volume was 10 μL. Compounds were analyzed in negative ionization mode. The MS was operated in Parallel Reaction Monitoring (PRM) mode with the resolution set to 60,000, AGC target 5e4, isolation window 4.0 *m*/*z*, and maximum IT 100 ms. The specific precursor and product ions, as well as the individual retention times, can be seen in Table 1. A collision energy setting of 18 was used for ZEN and ZEN-d6; 22 was used for all other compounds. These values were chosen to produce some fragmentation for structure confirmation while still giving a large precursor ion. To increase the number of data points in each analyte peak, the parent ions were monitored only within windows of time around the expected retention time-the resulting chromatogram can be seen in Fig. 2. Data acquisition and processing was carried out with Xcalibur (v.4.0) software. Additional MS conditions are provided in Supplemental Table 1.

**Table 1:** Retention times and corresponding precursor and product ions for ZEN and its metabolites and 2 mycoestrogen ISs.

	RT (min)	CE (eV)	Precursor Ion [M − H]‾	Product Ions
***ZEN***	6.85	18	317.1395	273.1497, 299.1288
***ZER***	6.29	22	321.1708	277.1809, 303.1602
***β-ZAL***	5.82	22	321.1708	277.1809, 303.1602
***α-ZOL***	6.40	22	319.1552	275.1654, 301.1446
***β -ZOL***	5.89	22	319.1552	275.1654, 301.1446
***ZAN***	6.86	22	319.1552	205.0866, 275.1654, 301.1446
***α-ZOL-d***_***7***_	6.40	22	326.1992	282.2092, 308.1885
***ZEN-d***_***6***_	6.85	18	323.1773	279.1874, 304.1604

Of note, the BetaSil™ phenyl hexyl column (100 × 4.6 mm, 3 μm) is no longer available. A 150 mm column with the same packing can be used with the same gradient as above if the re-equilibration period is extended by 1 min (total run time 16 min). With this column, the compounds elute about 1.5–1.6 min later.

### 2.6 Data analysis

To improve sensitivity, the Xcalibur quantitation method used an area sum of the product and precursor ions (Table 1). The mass tolerance in the quantitation method was set to 10 mmu, except for the internal standards, for which the setting was 5 mmu due to background interference. The mean peak area for the buffer blank was subtracted from the areas of the analyte peaks. In addition, a value equal to 0.17% of the corresponding ZEN peak area was subtracted from the areas of the ZAN peaks to correct for overlap of the ZEN [M+2] isotope. To generate the matrix-matched standard curves, the peak area ratio (area of analyte/area of IS) for the standard curve samples was plotted against the concentration of the standard. This standard addition approach was used to correct for endogenous levels of analytes in the tissue used for the standard curve: the endogenous level was calculated as the y-intercept divided by the slope. This value was added to the concentration of the standard to obtain the actual concentration, and the graph of peak area ratio vs. the corrected concentration was used to calculate the analyte concentrations in the samples. Values were also adjusted for the mass of the sample.

## 3 Results and discussion

### 3.1 Optimization of sample extraction protocol

Our lab previously used two different sample preparation protocols for the extraction of ZEN and its metabolites from biological matrices, both of which involved an SPE step. The first method, based on Hong et al., 2007, was used for analysis of ZEN mycoestrogens from solid tissue samples; this method involved liquid-liquid extraction (LLE) with MTBE followed by SPE on Sep-Pak silica columns [31,34]. The second method, which had been used for analysis of zeranols from urine, involved SPE on Chem Elut columns followed by another SPE on NH_2_ columns [21]. The efficacy of each method was compared using placental tissue to determine which provided better sensitivity and recoveries for the final protocol. When tested on 100 mg placenta samples spiked with mycoestrogen standards, the two-step SPE method gave standard curves with comparable linearity (R^2^ > 0.994) but better signal-to-noise ratios than the LLE method, indicating better sensitivity. Recoveries with the two-step SPE method were good for all compounds (86–91%), whereas the LLE method resulted in recoveries greater than 120% for β-ZAL, β-ZOL, and ZER. Therefore, the two-step SPE method was selected for the final protocol and optimized for placenta tissue. Endogenous analyte concentration levels were essentially the same for both methods.

### 3.2 Effect of placental tissue mass on extraction efficiency

Placenta tissue was homogenized in buffer, and aliquots corresponding to different tissue masses (100, 200, 300, and 400 mg) were spiked with different concentrations of the standards (0, 0.01, 0.05, 0.25 pg/mg tissue). The samples were processed as described in *Materials and Methods: Sample Preparation*, and the data was graphed as peak area ratio vs. concentration of the added analytes (Supplemental Fig. 1). All masses produced linear relationships with comparable R^2^ values ranging from 0.911 to 0.999. The 100 mg samples had the smallest R^2^ values overall, indicating a noisier linear relationship in comparison to the other sample masses. The 400 mg samples clogged the Chem Elut columns, slowing solvent flow and lengthening the process considerably. The 200 and 300 mg samples clogged the columns less and produced data with overall better R^2^ values. The regressions from the 200 mg samples demonstrated greater sensitivities than those from the 300 mg samples. Therefore, tissue samples in the 200–300 mg range were used for further optimization studies.

### 3.3 Elution solvent volume

The optimal solvent volume for eluting analytes from the Chem Elut columns was determined using both 200 mg and 300 mg tissue samples. Eluates were collected after 6, 9, 12, and 15 mL of MTBE had been added. On average, 60% of the total analytes and internal standards recovered were contained in the first 6 mL of the eluate. Later volume fractions still had substantial amounts of analytes, with an average of 14% of the total recovered fraction found between 12 and 15 mL. For this reason, 15 mL was chosen as the final volume of eluent used for the initial SPE step.

### 3.4 LCMS optimization

The LCMS method was based on an earlier method modified for an Orbitrap MS [31]. The higher resolution available on this instrument made it possible to better differentiate ions with similar masses; a detailed comparison of various instruments had previously been conducted by our group [35]. The original method also relied on an Atmospheric Pressure Chemical Ionization (APCI) source for ion creation. For this protocol, a HESI-II heated ESI source was selected as it gave larger peaks on the Orbitrap than the APCI source, often with higher signal-to-noise values, indicating greater sensitivity.

### 3.5 Method validation

Linearity was determined by calculating R^2^ values for the standard curves for each batch; the means are shown in Table 2. The standard curves were linear over a range of 0.0025–0.25 pg/mg tissue. In two additional experiments, the linear range was extended to 1 and 1.5 pg/mg tissue; the R^2^ values for all compounds remained excellent (≥0.997) for all experiments. Analyses of covariance showed that the slopes of the standard curves were not consistent among batches; therefore, a full standard curve was run with each batch.

**Table 2:** Performance data for the six mycoestrogens. LOD and LOQ values are the means of three determinations; recovery and R^2^ values are the means of thirteen batches. Accuracy and precision were determined using samples spiked with 0.05 pg/mg mycoestrogen standard mixture.

	LOD (pg/mg tissue)	LOQ (pg/mg tissue)	Accuracy (%)	Precision (%)	Recovery (%) (range)	R^2^
***ZEN***	0.0082	0.021	3.5	5.0	80 (51–116)	0.998
***ZER***	0.0051	0.013	4.6	5.8	68 (47–89)	0.998
***β-ZAL***	0.0055	0.014	5.5	7.9	73 (52–101)	0.996
***α-ZOL***	0.011	0.03	1.9	6.6	76 (37–92)	0.998
***β -ZOL***	0.0066	0.019	6.7	5.7	72 (56–91)	0.994
***ZAN***	0.0059	0.015	0.6	4.7	77 (48–102)	0.998

Limits of detection (LOD) were determined from a batch of 4–6 aliquots of tissue spiked with standards (0.01 pg/mg tissue). The detection limit was calculated as the standard deviation of the measured values of the analytes in the samples times the Student's t value for α = 0.01 (1-tailed) and n-1° of freedom [36]. The limit of quantitation (LOQ) was 10× the standard deviation. These values are shown in Table 2. LODs and LOQs for all analytes ranged between 0.0055 and 0.011 pg/mg and 0.013–0.03 pg/mg respectively, which were lower than those previously reported in the literature. Bernhoft et al., 2001 calculated LODs for ZEN (0.7 ng/g), α-ZOL (0.4 ng/g), and β-ZOL (3 ng/g) in placental and fetal tissues of Sprague Dawley rats [30]. Only one other study examined mycoestrogens in human placenta, through ex vivo perfusion, and reported LODs of 2 ng/g for α-ZOL, β-ZOL, α-ZAL, β-ZAL, ZAN and 0.6 ng/g for ZEN [22].

Precision and accuracy were determined by analyzing tissue spiked with the standards (equivalent to 0.05 pg/mg tissue); there were five replicate aliquots prepared per concentration, all on the same day. Accuracy was calculated as the difference between the theoretical and measured concentration of the compound expressed as a percent; precision was the relative standard deviation (RSD) for the replicate samples. Accuracy and precision values were below 10% for all compounds at 0.05 pg/mg. The results are shown in Table 2.

Recovery was calculated by comparing the peak areas of a sample of matrix spiked with standards (0.5 ng/mL) at the same time as the addition of the internal standards (before incubation/deconjugation) with a sample spiked just before the final drying step. Values were calculated for each of 13 batches of samples; the average values are shown in Table 2. Average recoveries for the six analytes ranged from 68% to 80% with relative standard deviations less than 20% for all analytes, indicating reasonably consistent recoveries among batches. A previous study utilizing a series of centrifugation steps reported slightly higher average extraction recoveries, ranging from 84% to 97% for human placenta spiked with a 15 μg/L mycoestrogen solution [22]. Bernhoft et al., 2001, whose work included the extraction of mycoestrogens from rat placental tissue using an immunoaffinity column, reported recoveries of 70%, which is comparable to our protocol [30].

### 3.6 Analysis of human placenta samples from the UPSIDE cohort

**Fig. 3:**
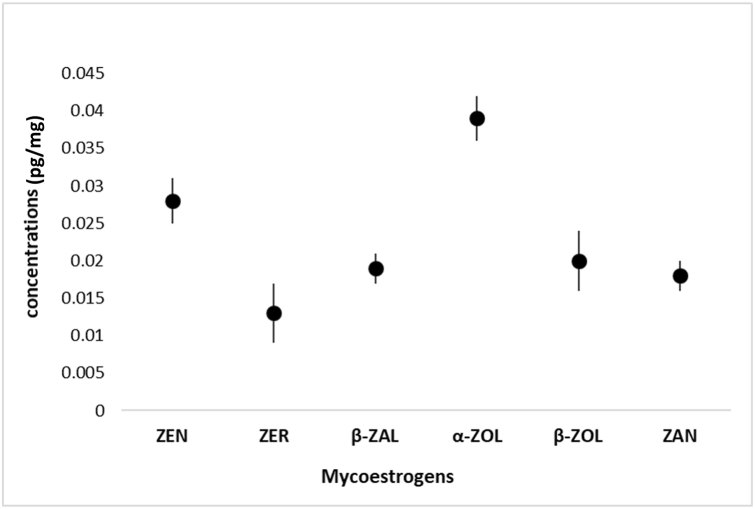
Median concentrations of mycoestrogens in placental tissue samples from the UPSIDE cohort (n = 25).

To further assess the effectiveness of the sample preparation method, we implemented the protocol for the quantitative analysis of mycoestrogens in a sub-sample of placentas (n = 25) collected from the UPSIDE birth cohort [33]. We report 68% detection of ZEN and 92% detection for at least one metabolite in these samples from Rochester, NY. The average concentration of the individual metabolites ranged from 0.003 to 0.012 pg/mg (Fig. 3), with the total mycoestrogen concentration (the sum of all metabolites) averaging 0.0123 pg/mg. We did not observe any associations between analytes in samples with multiple mycoestrogens present; however, ongoing work includes the expansion of biological analysis from the UPSIDE cohort and the exploration of compound correlations.

To our knowledge, there are no other previously published cohort studies analyzing mycoestrogen concentrations in placenta. The only study we found utilizing human placenta for mycoestrogens analysis does not represent the tissue's natural state, introducing mycoestrogen concentrations after tissue collection and focusing on the movement of ZEN across the placental barrier and its subsequent transformation into phase I and II metabolites [22]. Similar cohort studies have been published analyzing different endocrine disrupting compounds in placenta tissue, but neither the pathways nor the endpoints are comparable to our study [37,38].

A few animal studies have quantified placental mycoestrogens. Bernhoft et al., 2001 treated Sprague Dawley rats with a single dose of ZEN (0.74 mg/kg b.w.) and found that after 24 h, the placental mycoestrogen concentrations were reported to be 1.8 ± 1.0 ng/g (ZEN), 0.7 ± 0.2 ng/g (α-ZOL), and <3.0 ± 0 ng/g (β-ZOL) [30]. However, they noted their selected ZEN dosage was approximately 10000× the Canadian estimated human daily intake. Lange et al., 2002 treated pregnant Dutch belted rabbits with a subcutaneous ZER implant (0.25 mg/kg b.w.), reporting placental concentrations ranging from 0.09 to 0.14 pg/mg [29]. However, we emphasize that a direct translation from animal models to human implications is highly limited, as most animal studies do not reliably predict human outcomes [39].

ZEN and its metabolites have commonly been measured in urine; overall, mycoestrogens levels in placental tissue were lower compared to levels previously reported in human urine. In two small studies of pregnant women in the U.S. (n = 30; range: 0.003–0.19 μg/L) [25] and Bangladesh (n = 20; ZEN range: 0.01–0.21 ng/mL) [40], mycoestrogens were detected in 37% and 100% of urine samples, respectively. We have previously reported a 78% detection frequency for mycoestrogens in 9–10 year old girls, with urinary levels reported between 0.04 and 22.3 ng/mL [21]. Worldwide, other studies have measured mycoestrogens in urine at picogram to microgram levels [[41], [42], [43], [44], [45]].

## 4 Conclusion

ZEN crosses the placenta in humans and can potentially affect the fetus. Measuring mycoestrogens in the placenta may better characterize fetal exposure compared to maternal urinary assessments. In this study, we optimized our mycoestrogen protocol to measure ZEN and its metabolites in human placental tissue. Protocol linearity was very high (R^2^ ≥ 0.994) while achieving lower LODs (range: 0.0055–0.0110 pg/mg) than previously reported [22]. We presented an application of this protocol using placental tissue from women enrolled in a birth cohort in Rochester, NY. At least one metabolite was detected in 92% of these samples. This analytical protocol has the potential to advance human mycoestrogen research, particularly with respect to fetal and child health outcomes.

## Author contribution statement

Abigail Lazofsky: analyzed and interpreted the data, wrote the paper.

Anita Brinker: conceived and designed experiments, performed the experiments, analyzed and interpreted the data, wrote the paper.

Ruby Gupta: conceived and designed experiments, performed the experiments, analyzed and interpreted the data, wrote the paper.

Emily Barrett: conceived and designed experiments, analyzed and interpreted the data, contributed reagents, materials, analysis tools or data, wrote the paper.

Lauren M. Aleksunes: conceived and designed experiments, analyzed and interpreted the data, contributed reagents, materials, analysis tools or data, wrote the paper.

Zorimar Rivera-Núñez: conceived and designed experiments, analyzed and interpreted the data, contributed reagents, materials, analysis tools or data, wrote the paper.

Brian Buckley: contributed reagents, materials, analysis tools or data, wrote the paper.

## Data availability statement

Data will be made available on request.

## Additional information

Supplementary content related to this article has been published online at [URL].

## Declaration of competing interest

We declare that none of the authors have any known competing financial interests or personal relationships that could have appeared to influence the work reported in this paper.
