# Supplementary material for: Optimized extraction and analysis methods using liquid chromatography-tandem mass spectrometry for zearalenone and metabolites in human placental tissue
Source: Heliyon. 2023 Jun 4;9(6):e16940. doi: 10.1016/j.heliyon.2023.e16940 (PMC10361036; doi:10.1016/j.heliyon.2023.e16940)
Supplement: Multimedia component 1 [file mmc1.docx]

**Supplemental Information**

Abigail Lazofsky^a^, Anita Brinker^a^, Ruby Gupta^1,b^, Emily Barrett^a,c,^, Lauren M. Aleksunes^a,d,e^, Zorimar Rivera-Núñez^a,c^, Brian Buckley^a^*

^a^ Environmental and Occupational Health Sciences Institute, Rutgers University, 170 Frelinghuysen Road, Piscataway, NJ, 08854, USA

^b^ Department of Environmental and Occupational Health and Justice, Rutgers School of Public Health, Rutgers University, 683 Hoes Lane West, Piscataway, NJ, 08854, USA

^c^ Department of Biostatistics and Epidemiology, Rutgers School of Public Health, Rutgers University, 683 Hoes Lane West, Piscataway, NJ, 08854, USA

^d^ Department of Pharmacology and Toxicology, Ernest Mario School of Pharmacy, 160 Frelinghuysen Road, Rutgers University, Piscataway, NJ 08854, USA

^e^ Rutgers Center for Lipid Research, New Jersey Institute for Food, Nutrition, and Health, Rutgers University, 61 Dudley Road, New Brunswick, NJ 08901, USA

*Corresponding author- [bbuckley@eohsi.rutgers.edu](mailto:bbuckley@eohsi.rutgers.edu)

**Contents**

Table S1 …………………………………………………………………………………………………………………………………………….………………… 3

Figure S1 …………………………………………………………………………………………………………………………………………….………………. 4

**Supplemental Table 1.** Additional optimized MS instrument parameters used for zeranols analysis.

| Sheath gas flow | 45 |
| --- | --- |
| Auxiliary gas flow | 15 |
| Sweep gas flow | 0 |
| Spray Voltage (kV) | 2 |
| Capillary Temperature (°C) | 320 |
| S-Lens RF | 55 |
| Auxiliary gas heater temperature (°C) | 400 |

**
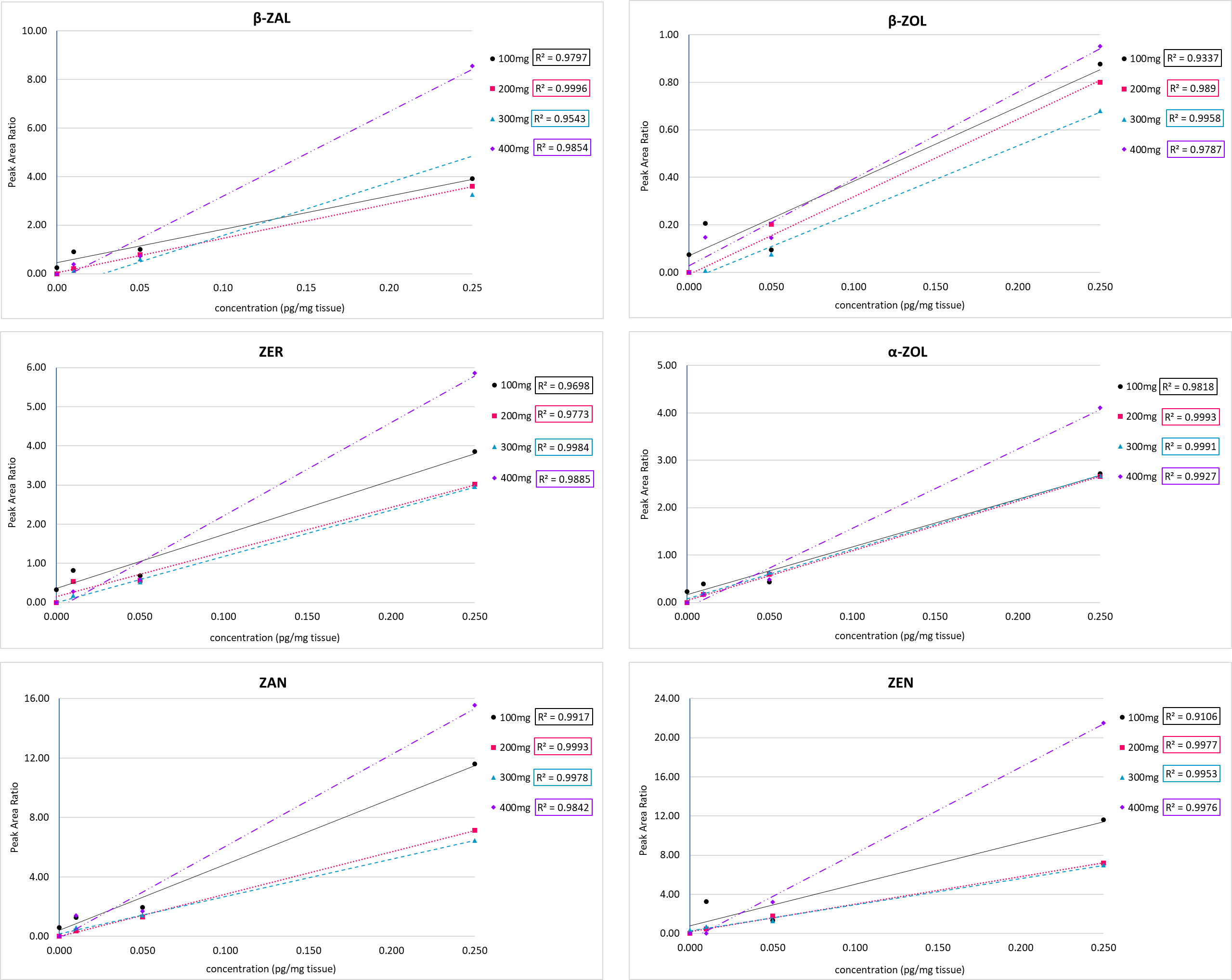
**

**Supplemental Figure 1.** Calibration curves created from spiking mycoestrogen standard mixture in 4 different masses of placental tissue to test the effect of placental mass on extraction efficiency. Based on resulting R^2^ values, slope of the calibration curve, and overall practicality, it was determined that between 200 and 300 mg of tissue was the most optimal mass for the developed extraction protocol.
